# Supplementary material for: Transcriptome Analysis Reveals the Effect of PdhR in Plesiomonas shigelloides
Source: Int J Mol Sci. 2023 Sep 23;24(19):14473. doi: 10.3390/ijms241914473 (PMC10572922; doi:10.3390/ijms241914473)
Supplement: Supplementary file 1 [file ijms-24-14473-s001.zip › ijms-2609506-supplementary.pdf]

Fig. S1

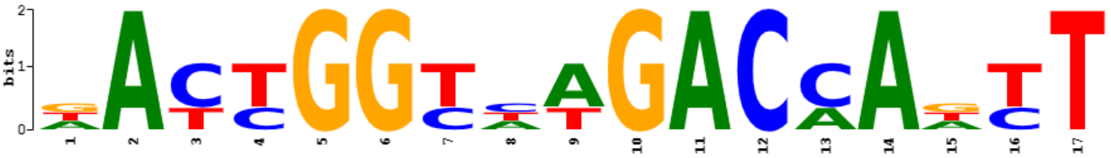

Fig. S1 The PdhR binding sites published on the PRODORIC website. The height of each letter presents the relative frequency of each base at different positions in the consensus sequence.

Fig. S2

pdhR	CGTTA**AATGGT**CTG**ACCAATT**TAAG
	CGTAAA**ATTGGT**AAT**ACCAATT**GACA
napF	CTTT**CGGGGT**ATT**ACCATTT**AACG
metR	CAATA**CATGGT**CAT**ACCAAG**CAAAA
nuoA	ATGAA**GTTGCT**TCT**ACCGATT**GTCCT
tfoX	AATT**ATTGCGT**GTT**ACAAATT**AAAG

Fig. S2 Potential PdhR binding sites (base marked in red) were predicted in the promoters of five genes, namely *pdhR*, n*apF*, *metR*, *nuoA*, and *tfoX*.

Fig. S3

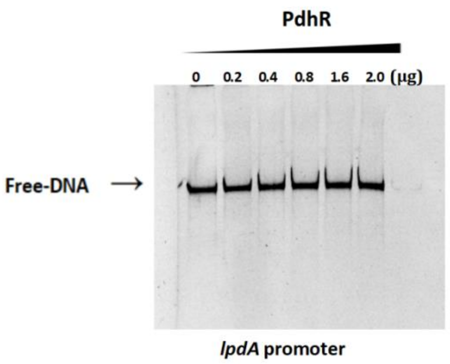

Fig. S3 The EMSAs between PdhR protein and the *lpdA* promoter.

Fig. S4

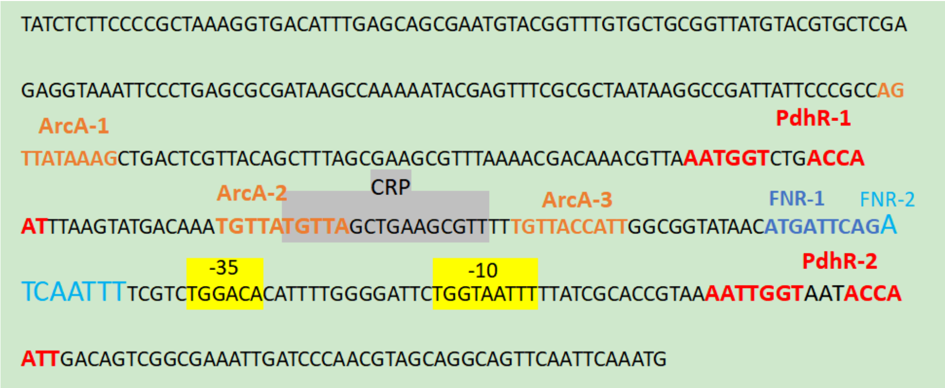

Fig. S4 PdhR, FNR, ArcA, and cAMP-CRP binding sites were predicted in the promoter of *pdhR* by the PRODORIC website.

Fig. S5

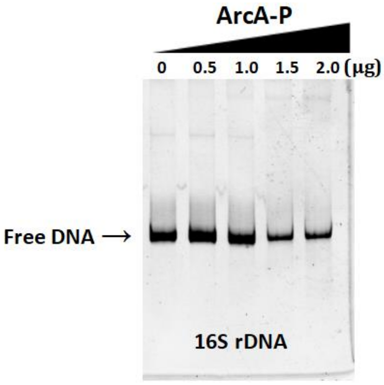

Fig. S5 The EMSAs between phosphorylated ArcA and 16S rDNA as a control.

Fig. S6

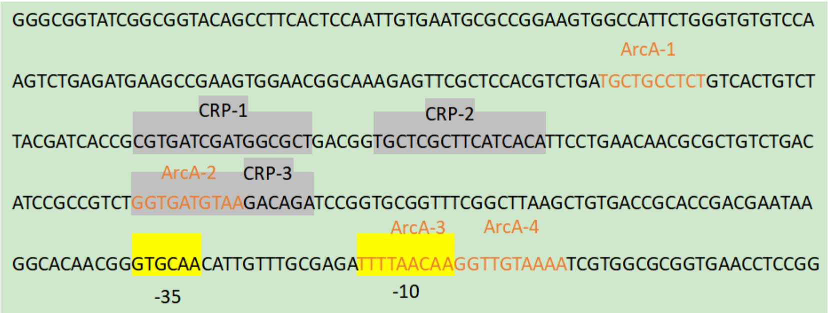

Fig. S6 -10 and -35 regions were predicted in the promoter of *lpdA* by the Softberry website.

Fig. S7

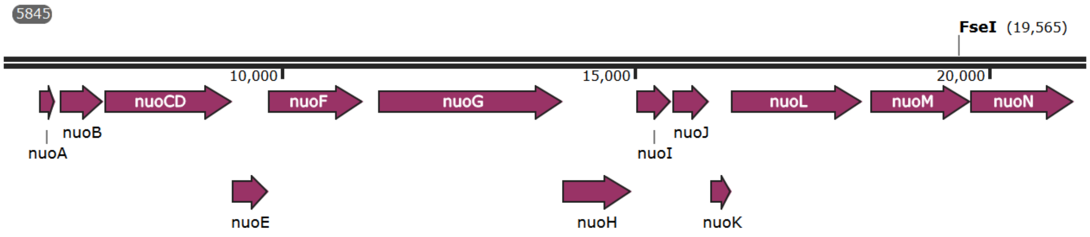

Fig. S7 NADH-quinone oxidoreductase (NDH-1) gene cluster (*nuoA-nuoN*) in the *P. shigelloides* genome.

Fig. S8

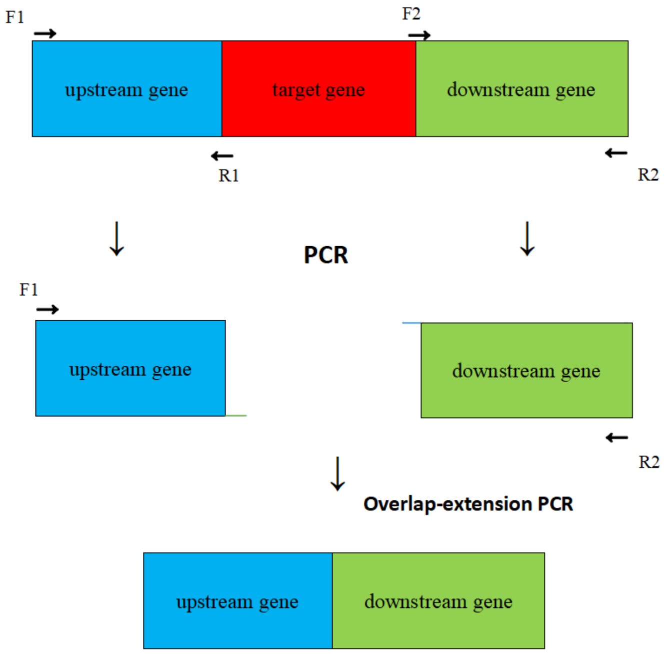

Fig. S8 Graphical process of the deletion of the target gene in this study. The genome was used as a template, and the upstream and downstream genes of the target gene were amplified by PCR using two pairs of primers F1 and R1, and F2 and R2, respectively. Furthermore, F1 and R2 contain the restriction sites carried by the pRE112 plasmid, and F2 and R1 contain reverse complementary sequences of 10 bases of each other in their respective 5' ends. Subsequently, the upstream and downstream genes were simultaneously used as templates and linked together by Overlap-extension PCR using F1 and R2. After that, the upstream and downstream ligation fragments and the pRE112 plasmid were reconstructed into new vectors by restriction enzyme digestion as well as T4 DNA ligase ligation, which was subsequently transferred to *E. coli* S17-1 λpir and conjured with the WT strain, and mutant strains successfully homologous recombinant were screened out by corresponding antibiotics.

Table S1 Bacterial strains and plasmids used in this study.

Strains/plasmids	Genotype or relevant characteristics[a]	Source or reference
Plesiomonas shigelloides strains		
G5884	Wild type, serotype O45:H2[b]; Amp^r	CNCTC[b] Aer 44/89
Δ*pdhR*	*pdhR* gene deletion mutant of G5884; Amp^r	This study
Δ*pdhR*/*pdhR*$^+$	the complementation of *pdhR* in *P. shigelloides*; Amp^r	This study
Δ*arcA*	*arcA* gene deletion mutant of G5884; Amp^r	This study
Δ*fnr*	*fnr* gene deletion mutant of G5884; Amp^r	This study
Δ*crp*	*crp* gene deletion mutant of G5884; Amp^r	This study
WT/lux	WT containing pMS402; Amp^r, Km^r	This study
WT/*flgB*-lux	WT containing pMS402 carrying *flgB* promoter; Amp^r, Km^r	This study
WT/*flgP*-lux	WT containing pMS402 carrying *flgP* promoter; Amp^r, Km^r	This study
WT/*flhA*-lux	WT containing pMS402 carrying *flhA* promoter; Amp^r, Km^r	This study
WT/*flaC*-lux	WT containing pMS402 carrying *flaC* promoter; Amp^r, Km^r	This study
WT/*fliM$_L$*-lux	WT containing pMS402 carrying *fliM$_L$* promoter; Amp^r, Km^r	This study

WT/*flgB$_L$*-lux	WT containing pMS402 carrying *flgB$_L$* promoter; Amp^r, Km^r	This study
WT/*flgD$_L$*-lux	WT containing pMS402 carrying *flgD$_L$* promoter; Amp^r, Km^r	This study
WT/*fliC*-lux	WT containing pMS402 carrying *fliC* promoter; Amp^r, Km^r	This study
WT/*fliK$_L$*-lux	WT containing pMS402 carrying *fliK$_L$* promoter; Amp^r, Km^r	This study
Δ*pdhR/flgB*-lux	Δ*pdhR* containing pMS402 carrying *flgB* promoter; Amp^r, Km^r	This study
Δ*pdhR/flgP*-lux	Δ*pdhR* containing pMS402 carrying *flgP* promoter; Amp^r, Km^r	This study
Δ*pdhR/flhA*-lux	Δ*pdhR* containing pMS402 carrying *flhA* promoter; Amp^r, Km^r	This study
Δ*pdhR/flaC*-lux	Δ*pdhR* containing pMS402 carrying *flaC* promoter; Amp^r, Km^r	This study
Δ*pdhR/fliM$_L$*-lux	Δ*pdhR* containing pMS402 carrying *fliM$_L$* promoter; Amp^r, Km^r	This study
Δ*pdhR/flgB$_L$*-lux	Δ*pdhR* containing pMS402 carrying *flgB$_L$* promoter; Amp^r, Km^r	This study
Δ*pdhR/flgD$_L$*-lux	Δ*pdhR* containing pMS402 carrying *flgD$_L$* promoter; Amp^r, Km^r	This study

Δ*pdhR/fliC*-lux	Δ*pdhR* containing pMS402 carrying *fliC* promoter; Amp^r, Km^r	This study
Δ*pdhR/fliK$_L$*-lux	Δ*pdhR* containing pMS402 carrying *fliK$_L$* promoter; Amp^r, Km^r	This study
WT/*pdhR*-lux	WT containing pMS402 carrying *pdhR* promoter; Amp^r, Km^r	This study
WT/*metR*-lux	WT containing pMS402 carrying *metR* promoter; Amp^r, Km^r	This study
WT/*nuoA*-lux	WT containing pMS402 carrying *nuoA* promoter; Amp^r, Km^r	This study
Δ*pdhR/pdhR*-lux	Δ*pdhR* containing pMS402 carrying *pdhR* promoter; Amp^r, Km^r	This study
Δ*pdhR/metR*-lux	Δ*pdhR* containing pMS402 carrying *metR* promoter; Amp^r, Km^r	This study
Δ*pdhR/nuoA*-lux	Δ*pdhR* containing pMS402 carrying *nuoA* promoter; Amp^r, Km^r	This study
Δ*arcA/pdhR*-lux	Δ*arcA* containing pMS402 carrying *pdhR* promoter; Amp^r, Km^r	This study
Δ*arcA/lpdA*-lux	Δ*arcA* containing pMS402 carrying *lpdA* promoter; Amp^r, Km^r	This study
Δ*fnr/pdhR*-lux	Δ*fnr* containing pMS402 carrying *pdhR* promoter; Amp^r, Km^r	This study

Δ*fnr/lpdA*-lux	Δ*fnr* containing pMS402 carrying *lpdA* promoter; Amp^r, Km^r	This study
Δ*crp/pdhR*-lux	Δ*crp* containing pMS402 carrying *pdhR* promoter; Amp^r, Km^r	This study
Δ*crp/lpdA*-lux	Δ*crp* containing pMS402 carrying *lpdA* promoter; Amp^r, Km^r	This study
pBAD33-*pdhR*-3×Flag	WT containing plasmid pBAD33-*pdhR*-3×Flag; Amp^r, Cm^r	This study
pBAD33-a*rcA*-3×Flag	Δ*arcA* containing plasmid pBAD33-pdhR-3×Flag; Amp^r, Cm^r	This study
E. coli strains		
DH5α λ*pir*	Transformation host	Lab collection
S17–1 λ*pir*	Tp^R Sm^R *recA*, *thi*, *pro*, *hsdR*-M$^+$RP4: 2-Tc:Mu: Km Tn7 λ*pir*, Km^r , Sm^r , Tp^r	Lab collection
BL21(DE3)	Host strain for protein expression	Lab collection
BL21/pET-*pdhR*	BL21 with pET28a carrying the *pdhR* gene; Km^r	This study
BL21/pET-*arcA*	BL21 with pET28a carrying the *arcA* gene; Km^r	This study
BL21/pMAL-c5X-*fnr*	BL21 with pMAL-c5X carrying the *fnr* gene; Amp^r	This study
BL21/pMAL-c5X-*crp*	BL21 with pMAL-c5X carrying the *crp* gene; Amp^r	This study
Plasmids		

pRE112	Widely used gene knocked vector, with onT RP4; Cm^r	Lab collection
pMS402	For construct promoter-luxCDABE reporter fusion; Km^r	Lab collection
pRE112-*pdhR*$^{-}$	pRE112 containing the homologous arms of *pdhR* gene of G5884; Cm^r	This study
pRE112-*pdhR*$^{+}$	pRE112 containing the homologous arms of *pdhR* and *pdhR* gene of G5884; Cm^r	This study
pRE112-*arcA*$^{-}$	pRE112 containing the homologous arms of *arcA* cluster of G5884; Cm^r	This study
pRE112-*fnr*$^{-}$	pRE112 containing the homologous arms of *fnr* cluster of G5884; Cm^r	This study
pRE112-*crp*$^{-}$	pRE112 containing the homologous arms of *crp* cluster of G5884; Cm^r	This study
pBAD33-pdhR-3×Flag	pBAD33 carrying the pdhR gene with 3×Flag; Cmr	This study
pBAD33-arcA-3×Flag	pBAD33 carrying the arcA gene with 3×Flag; Cmr	This study
pMS402-*flgB*	pMS402 with *flgB* promoter; Km^r	This study
pMS402-*flgP*	pMS402 with *flgP* promoter; Km^r	This study
pMS402-*flhA*	pMS402 with *flhA* promoter; Km^r	This study
pMS402-*flaC*	pMS402 with *flaC* promoter; Km^r	This study
pMS402-$fliM_L$	pMS402 with $fliM_L$ promoter; Km^r	This study
pMS402-$flgB_L$	pMS402 with $flgB_L$ promoter; Km^r	This study
pMS402-$flgD_L$	pMS402 with $flgD_L$ promoter; Km^r	This study

pMS402-*fliC*	pMS402 with *fliC* promoter; Km^{r}	This study
pMS402-*fliK*$_{L}$	pMS402 with *fliK*$_{L}$ promoter; Km^{r}	This study
pMS402-*pdhR*	pMS402 with *pdhR* promoter; Km^{r}	This study
pMS402-*metR*	pMS402 with *metR* promoter; Km^{r}	This study
pMS402-*nuoA*	pMS402 with *nuoA* promoter; Km^{r}	This study
pMS402-*lpdA*	pMS402 with *lpdA* promoter; Km^{r}	This study

[a] r = resistant.

[b] CNCTC, Czech National Collection of Type Cultures, the Czech Republic.

Table S2 Primers used in this study

Name	Sequence (5′–3′)
Primers for construction of mutants	
Δ*pdhR*-F1	GCTCTAGATTATTTATTCCTATCTCTTCCCCG
Δ*pdhR*-R1	AAACGCCTTCTGCCTGCTACGTTGGGATC
Δ*pdhR*-F2	GTAGCAGGCAGAAGGCGTTTTTCCGTCAG
Δ*pdhR*-R2	GGGGTACCTCCCTGGAGAGATGTGACCC
Δ*arcA*-F1	GCTCTAGATTTTGGCTAAATCTGTGTGC
Δ*arcA*-R1	GGTCAATTGCGTGGGCCAACTGCTTGCGCCTTC
Δ*arcA*-F2	GAAGGCGCAAGCAGTTGGCCCACGCAATTGACC
Δ*arcA*-R2	GGGGTACCTTTGAGAAGGTGATGCCG

Δ*fnr*-F1	GGGGTACCTACTGGCCGCCGTGATG
Δ*fnr*-R1	CCCAGATAGAACAAAGTGTGAGCTTAACGTATAACACTACCTGACATTGAT
Δ*fnr*-F2	TAACGTATAACACTACCTGACATTGATAGCTCACACTTTGTTCTATCTGGG
Δ*fnr*-R2	CGAGCTCATCGTGCCCGCCTTGAAT
Δ*crp*-F1	GGGGTACCAAAGGGAAGATTTTTAGCAGTGC
Δ*crp*-R1	CAAGCGACTATGTAGAGGAAATATAAAATCTCCGGTTACTTTTCCGGT
Δ*crp*-F2	ACCGGAAAAGTAACCGGAGATTTTATATTTCCTCTACATAGTCGCTTG
Δ*crp*-R2	GCTCTAGAGGTGATCCACTTTCGCGAA
Primers for identification of plasmid	
pRE112-F	CACTGTTCGTCCATTTCCG
pRE112-R	TTCGTCTCAGCCAATCCCT
pBAD33-F	AACAAAGCGGGACCAAAG
pBAD33-R	AGAGCGTTCACCGACAAA
pMS402-F	GGTCAAATGAATGCAGGGCT
pMS402-R	AGAGTCATTCAATATTGGCAGGT
pMAL-c5X-F	GGTGAAATCATGCCGAACATCC
pMAL-c5X-R	TCGCAACGTTCAAATCCGCT
Primers for construction of complemented strain	
Δ*pdhR*-F1	GCTCTAGATTATTTATTCCTATCTCTTCCCCG
Δ*pdhR*-R2	GGGGTACCTCCCTGGAGAGATGTGACCC
Primers for lux	
lux-*flgB*-F	CCGCTCGAGTTTTGTTTCGGTCGCAGGTG
lux-*flgB*-R	CGGGATCCACTTAATGCCAAGACACCGC

lux-*flgP*-F	CCGCTCGAGCGTAACGTTAGGGTAAAGCGG
lux-*flgP*-R	CGGGATCCGTAATGACATTGACACTCCGGC
lux-*flhA*-F	CCGCTCGAGGCAAGCTGGCATCTCTGTAC
lux-*flhA*-R	CGGGATCCCTATCCTCTGTCTACCGCGC
lux-*flaC*-F	CCGCTCGAGATTTCTTGACATGCCGCGTT
lux-*flaC*-R	CGGGATCCATCTCCGTTAAACTTGCCGC
lux-*fliM_L*-F	CCGCTCGAGGCAACGTCTTACCGGGTCTT
lux-*fliM_L*-R	CGGGATCCGCTGTGTGATACATGACGCTGA
lux-*flgB_L*-F	CCGCTCGAGCCTCGCTCAGGCGCTG
lux-*flgB_L*-R	CGGGATCCGTCCCCCTGCAAACAACG
lux-*flgD_L*-F	CCGCTCGAGGAACATGATGATGCAAGCTGG
lux-*flgD_L*-R	CGGGATCCAATCTATCCTCTGTCTACCGCG
lux-*fliC*-F	CCGCTCGAGGATTGAGCGTCAGCTGCAAG
lux-*fliC*-R	CGGGATCCCTTGTTACTGGTATTAGGCGGAA
lux-*fliK_L*-F	CCGCTCGAGCGGAAGTGATTGACGGACAG
lux-*fliK_L*-R	CGGGATCCCTCAGTGTAAAAACACCCGACC
lux-*pdhR*-F	CCGCTCGAGCGAGAGGTAAATTCCCTGAGC
lux-*pdhR*-R	CGGGATCCTGAATTGAACTGCCTGCTACG
lux-*metR*-F	CCGCTCGAGACATTGCCCACCATCAAACT
lux-*metR*-R	CGGGATCCTCCCTGACCAAATAAGGCTCA
lux-*nuoA*-F	CCGCTCGAGCGTGGCTGATGAAGTTGCTT
lux-*nuoA*-R	CGGGATCCGGCGAGCACTACCTACAGAA
lux-*lpdA*-F	CCGCTCGAGCGGCGGTACAGCCTTCAC
lux-*lpdA*-R	CGGGATCCCGCCACGATTTTACAACCTT

Primers for qRT-PCR	
gyrB-RT-F	GATTTGCGCACTGGGTAGCC
gyrB-RT-R	GCGGCTGTTTGGATCCATGG
pdhR-RT-F	ATCTGGTGGTGGTGAGTGAG
pdhR-RT-R	TAAAGGCCAAATGACGGTGC
aceE-RT-F	TGGGTTCTGGCGCTATCCTG
aceE-RT-R	TCACAGCAGTCACGAACGGT
aceF-RT-F	AGCGTCTGGAATCCGGTCAG
aceF-RT-R	TGGATCCGACCCAGCTCAAC
lpdA-RT-F	GGTGGTGAATGGTCTGGGTA
lpdA-RT-R	TCCAGACCGATAATACCGCC
sdhC-RT-F	TGTTGTCCCTGTCGCTCTCC
sdhC-RT-R	GCCGTCAAAATGCCCCACAA
sdhD-RT-F	TGACGTACGGGATCTGGAAG
sdhD-RT-R	ACCAACACCACCACAAACTG
sdhA-RT-F	CAGCGCTGCAGATTTCCCAG
sdhA-RT-R	CGATGGCATTTTGGTCCCCG
sdhB-RT-F	TTTGATTGTCGACATGGGGC
sdhB-RT-R	GTATGCTGCCAGTAAACCGG
psrA-RT-F	CCCCAAGTTTGCAGCAGGTC
psrA-RT-R	CCGCACGAGTGAATAACGCC
metR-RT-F	TCCCGTACCCAAACAGCGTT
metR-RT-R	TGCCGCCACCATTTGCAATT
tfoX-RT-F	GCCCGCTTTATTGCACGAGG

tfoX-RT-R	GCTTTGCTAAACCACCGCGT
nuoA-RT-F	TCAGGGAGAGCGGTTGGTTG
nuoA-RT-R	GGTCCAATCCAGTGCCCCAA
nuoB-RT-F	TGGGCAAGCTGGAGGATGTT
nuoB-RT-R	CTTGACGCGGAGATGCCCTA
cysD-RT-F	GATTCAATGGCGCCGGTCAG
cysD-RT-R	CGACGGCATGCTGATGATGG
cysJ-RT-F	GATTCAATGGCGCCGGTCAG
cysJ-RT-R	CGACGGCATGCTGATGATGG
moaB-RT-F	GCTATTTTGGTCACCGGCGG
moaB-RT-R	CCAGTGCACGCGATTGGATC
moaE-RT-F	ATTGCCGCCGAATACACGTG
moaE-RT-R	GCCATTAACGCTGCCTTGCT
modB-RT-F	CTTACGACTGGCCGCGACTA
modB-RT-R	GAGCGAGGAGAGAAGGCCAG
modC-RT-F	CGTTTACTGCTGCGCGTGAT
modC-RT-R	CCATCACCATGCCCTGTTGC
ntrC-RT-F	TCATCATGACCGCCCACTCC
ntrC-RT-R	AAGGCGCGCTCAACTAATGC
napF-RT-F	GACCAGTTTATCGCGGCGTC
napF-RT-R	GCAACGCGTACACTGATCGG
napA-RT-F	ATGTGGGTGGAGAAGGAAGG
napA-RT-R	CAGGCCAAACTTCATCCACC
napG-RT-F	GCCGTGCGAGATGTGTGATG

napG-RT-R	CATGATCCAGCAACACCGCC
phlA-RT-F	AAGATCACCTACAGCGCCCC
phlA-RT-R	GGCGGCAGTCAGGGTCATAT
phlB-RT-F	ATGCCGACCTGTACCGCTAC
phlB-RT-R	CGATGAAAAGCCACGGACGG
flgO-RT-F	GCACTCTCCGGGCTGTTATT
flgO-RT-R	ATCAGATTGCCCAGCCAGTT
flgP-RT-F	CGGGTGTTAGAGCGTCAACT
flgP-RT-R	AACGAGTTCAGACCGGTGAC
flgM-RT-F	GCGCTGGATCCTCTGATTGA
flgM--RT-R	TCATCACTTCGCCCAGCAAT
cheV-RT-F	AGCGCACACAATTAGTCGGA
cheV-RT-R	CAATCGCCAAGCTCATGTCG
flgB-RT-F	ACCGAAATGCTGGCCAGTAA
flgB-RT-R	CTGTCGGGCTGATTGGGAAT
flgK-RT-F	GGCTGAGTACACCCAAGTCT
flgK-RT-R	CGCATTTCAAACATCGCACC
flaC-RT-F	TTGCGATCGACTCATCCCTG
flaC-RT-R	CAGGATCTGCTGCTTGGTCA
fliE-RT-F	AGTGCTTTGGACTTCTCTGCT
fliE-RT-R	TACCTGTACCGTCGCCTCAA
flhA-RT-F	TACGCTGTGGGTATTGTGGT
flhA-RT-R	TCCATGGAGCCGTAGAAGTC
cheY-RT-F	ATACCCACGAAGCGGATGAC

cheY-RT-R	TCTGTTCACGCTTGGCTTCT
motA-RT-F	CCGCTCGAGTCCCCAGGTCTCAAAATCGT
motA-RT-R	CGGGATCCCATCAAACCTCTGTGCTCGT
$fliM_L$-RT-F	AATTTGATGACTGCTGGCGC
$fliM_L$-RT-R	CAGACCGTTAAATGGCAGCG
$fliH_L$-RT-F	CAGCAAGTGTTCCATCAGCA
$fliH_L$-RT-R	CAACTGCCACTTGTTCACCA
$fliJ_L$-RT-F	TGGAATTGTTGCAGCAGGTC
$fliJ_L$-RT-R	CTTTTGCAGCAAGGTATCGC
$flgB_L$-RT-F	GCCGAACCGAATTATGTGGC
$flgB_L$-RT-R	TTGCTTGCTTTAACCCGCTG
$flgH_L$-RT-F	CAGCGATGGAAAGGGACAAG
$flgH_L$-RT-R	GGTGTTGCTCTCTTTGCCAA
$flgK_L$-RT-F	TTGAAAGACCCAGCCGATCT
$flgK_L$-RT-R	GTCTGAATCGCCCAATCACC
$fliC_L$-RT-F	GTGCCCAAACAACAACAGCA
$fliC_L$-RT-R	CACCCAGCTGTGCCATAGAT
lafB-RT-F	AAAAGCTTCGTCACCGCCTA
lafB-RT-R	ACCTGACCTACACTGTTGCG
$fliA_L$-RT-F	CCGCTCGAGGCAAGCTGGCATCTCTGTAC
$fliA_L$-RT-R	CGGGATCCCTATCCTCTGTCTACCGCGC
sipA-RT-F	TTGACTCGACATTCTGCGCT
sipA-RT-R	CAAATCCGGCCACAAAGCTC

sipD-RT-F	GCCTGCCACGCTTGAAATTT
sipD-RT-R	TCAGTGATCGGAAGGTTGCC
sipB-RT-F	GCCTCAAGCGCAGACTTACT
sipB-RT-R	CTGGGGCAATTGATGGTCCT
spaS-RT-F	CAACCACACCAGTAGACGCA
spaS-RT-R	AACGGATGTGAAATTGGCGC
spaT-RT-F	GCTTCCGCTTCATCGAGTCT
spaT-RT-R	GAAATGGTCTGGGAAGCGGT
spaR-RT-F	TTCGCTGGATCAATCGTGCT
spaR-RT-R	GTCATGCTGGGCCTGTTACT
spaQ-RT-F	GTTTCACCATACCAGCCCGA
spaQ-RT-R	ATGATTTGATGTTTGCGGGCAA
spaN-RT-F	GCTGCTGATGTGGATTTCGC
spaN-RT-R	CAGCCTTCAGATTCGGTGGT
invA-RT-F	AGTTAACCACGCGCACCATA
invA-RT-R	TCAAGGGCCACTGGAAAAGG
invE-RT-F	CCAACACCCGCTCAAAACTG
invE-RT-R	CAGCGTTTCGTTCAGTCTGC
invG-RT-F	CGCCCATCCAGAGTGCTTAT
invG-RT-R	GAGGATCTGGAGCAACTCGG
hilA-RT-F	TACAAAATCGCATTGCCGCC
hilA-RT-R	AGCTGCAGTACAATGACGCT

prgJ-RT-F	ATTACACGCCAAGACACCGAT
prgJ-RT-R	TCGAGGCGAGCTAAGATTGC
prgK-RT-F	AGTCGCCCAGATGTTTCCTG
prgK-RT-R	ACGCGGACAAGTGAATGGAT
16S-RT-F	GGCTCTTCGGACCTCATGCT
16S-RT-R	TTCTTCACACACGCGGCATG
rpoS-RT-F	TTCTCCACCGCCCGAATCAA
rpoS-RT-R	TGCGCAACGTGTTCTGGATG
Primers for EMSAs	
pdhR-EMSA-F	TCCCCGCTAAAGGTGACATT
pdhR-EMSA-R	TTGGGATCAATTTCGCCGAC
metR-EMSA-F	ACCAAGCAAAATCACCCACC
metR-EMSA-R	TCCCTGACCAAATAAGGCTCA
nuoA-EMSA-F	CGTGGCTGATGAAGTTGCTT
nuoA-EMSA-R	GGCGAGCACTACCTACAGAA
tfoX-EMSA-F	AAAGTTGCTTAATTGGTTGCA
tfoX-EMSA-R	GCTCCCCTCCATAACTGCTT
napF-EMSA-F	AAGCGCACTCTTTCAACGTG
napF-EMSA-R	GCATGCTCCTATGACACAGC
lpdA-EMSA-F	ACTCCAATTGTGAATGCGCC
lpdA-EMSA-R	GCGCCACGATTTTACAACCT
16s-EMSA-F	AGAGTTTGATCATGGCTCAGATT
16s-EMSA-R	ACGTCAATGCCACTAGGTATTAACT

Primers for protein cloning	
pMAL-*pdhR*-F	ACGCGTCGACATGGATGTTTGCATGGCCT
pMAL-*pdhR*-R	CCCAAGCTTTCAATCCCTGCGTTGCTG
pMAL-*crp*-F	CGGGATCCATGGTTCTTGGTAAACCGCAA
pMAL-*crp*-R	GGAATTCTTAACGCGTGCCGTACACC
pET-*fnr*-F	CGGGATCCATGATCCCAGAGAAAAAGACGATA
pET-*fnr*-R	CGGAATTCTTAGGCTTGTTTATTATTACCGGC
pET-*arcA*-F	CGGGATCCATGCAAACCCCGCACATT
pET-*arcA*-R	CCCTCGAGTTACTCTTCCAGCTCGCCG
Primers for ChIP	
pBAD33-*pdhR*-3×Flag-F	GGGGTACCATGCAAACCCCGCACATT
pBAD33-*pdhR*-3×Flag-R	ACGCGTCGACTTACTCTTCCAGCTCGCCG
pBAD33-*arcA*-3×Flag-F	GGGGTACCATGGATGTTTGCATGGCCT
pBAD33-*arcA*-3×Flag-R	ACGCGTCGACTCAATCCCTGCGTTGCTG

Underlined letters show Kpn1、sacI、Xba1、BamhI、EcoRI or XhoI restriction site.
F/R: The upstream and downstream primers

Table S3 Transcriptome revealed differential expressed genes related to PdhR of the *P. shigelloides*

Gene id	Gene name	log2FoldChange	pvalue
NODE_1_length_84377_cov_94.352280-orf00009	-	-5.750207073	0.040964585
NODE_1_length_84377_cov_94.352280-orf00044	-	-1.657099684	0.045435959
NODE_146_length_604099_cov_50.948364-orf00073	-	-1.976992473	0.009732694
NODE_146_length_604099_cov_50.948364-orf00246	-	-1.527239869	0.000761809
NODE_146_length_604099_cov_50.948364-orf00293	-	-2.000598368	0.016492881
NODE_146_length_604099_cov_50.948364-orf00319	-	-2.133682897	0.014485426
NODE_146_length_604099_cov_50.948364-orf00360	*feoA*	-1.219917474	0.042571852
NODE_146_length_604099_cov_50.948364-orf00384	-	-1.103668089	0.008708373
NODE_146_length_604099_cov_50.948364-orf00486	*motB*	-1.794287053	0.000178258

NODE_146_length_604099_cov_50.948364-orf00487	-	-2.890662604	5.15E-07
NODE_146_length_604099_cov_50.948364-orf00551	*pcm*	-1.203117948	0.011314746
NODE_146_length_604099_cov_50.948364-orf00633	-	-6.326207331	0.004174171
NODE_185_length_341402_cov_46.264301-orf00062	*fruK*	-1.053623998	0.011632589
NODE_185_length_341402_cov_46.264301-orf00119	-	-2.000598368	0.016492881
NODE_185_length_341402_cov_46.264301-orf00122	-	-1.328464314	0.002489721
NODE_185_length_341402_cov_46.264301-orf00194	*hybC*	-1.024901888	0.017533811
NODE_2_length_96615_cov_81.982597-orf00021	*iacP*	-1.392063456	0.009693408
NODE_2_length_96615_cov_81.982597-orf00022	*sipA*	-1.924520502	9.44E-06
NODE_2_length_96615_cov_81.982597-orf00023	*sipD*	-1.83844156	3.64E-05
NODE_2_length_96615_cov_81.982597-orf00024	*sipC*	-1.987433636	4.04E-06
NODE_2_length_96615_cov_81.982597-orf00025	-	-2.825130344	3.30E-07
NODE_2_length_96615_cov_81.982597-orf00026	*sipB*	-2.765008618	6.76E-10
NODE_2_length_96615_cov_81.982597-orf00027	*spaT*	-2.927886667	3.30E-10
NODE_2_length_96615_cov_81.982597-orf00032	*spaP*	-1.27545036	0.008594401
NODE_2_length_96615_cov_81.982597-orf00036	-	-2.245008965	0.001111067
NODE_2_length_96615_cov_81.982597-orf00039	*invA*	-1.224115715	0.006805885
NODE_2_length_96615_cov_81.982597-orf00040	*invE*	-1.53865129	0.001381097
NODE_2_length_96615_cov_81.982597-orf00041	*invG*	-1.572946827	0.000437011
NODE_2_length_96615_cov_81.982597-orf00042	*invF*	-2.197459603	3.09E-06
NODE_2_length_96615_cov_81.982597-orf00048	*hilA*	-1.642490729	0.000391751
NODE_2_length_96615_cov_81.982597-orf00049	*iagB*	-1.231936286	0.021782468
NODE_2_length_96615_cov_81.982597-orf00050	-	-2.132694539	3.40E-06
NODE_2_length_96615_cov_81.982597-orf00051	*prgH*	-2.127195052	4.64E-06
NODE_2_length_96615_cov_81.982597-orf00052	*prgI*	-1.727208858	8.12E-05
NODE_2_length_96615_cov_81.982597-orf00053	*prgJ*	-2.096057454	1.97E-05
NODE_2_length_96615_cov_81.982597-orf00054	*prgK*	-1.687186935	0.000167784
NODE_2_length_96615_cov_81.982597-orf00055	*orgA*	-1.646968018	0.003514632
NODE_217_length_118346_cov_49.856361-orf00015	-	-1.430579981	0.000866015
NODE_217_length_118346_cov_49.856361-orf00128	-	-1.183875389	0.027595171
NODE_26_length_137541_cov_47.023979-orf00021	-	-1.104502306	0.012259875
NODE_26_length_137541_cov_47.023979-orf00039	-	-5.750207073	0.040964585
NODE_3_length_124211_cov_75.007332-orf00052	-	-1.4345977	0.001578183
NODE_46_length_93402_cov_46.306866-orf00080	-	-1.658804788	0.000263132
NODE_46_length_93402_cov_46.306866-orf00090	*yfiQ*	-1.366579597	0.011094044
NODE_59_length_176703_cov_50.362434-orf00075	*ntrC*	-1.059796397	0.025927384
NODE_59_length_176703_cov_50.362434-orf00103	-	-4.422222292	1.71E-17
NODE_59_length_176703_cov_50.362434-orf00106	*bglB*	-1.266454279	0.003249942
NODE_59_length_176703_cov_50.362434-orf00189	-	-1.037536327	0.012499403
NODE_59_length_176703_cov_50.362434-orf00217	-	-1.327199334	0.001746566
NODE_6_length_63404_cov_68.699402-orf00009	*mgtE*	-2.135819427	0.003489554
NODE_6_length_63404_cov_68.699402-orf00020	-	-1.539523247	0.034035355
NODE_6_length_63404_cov_68.699402-orf00036	-	-3.267816144	0.021566548
NODE_61_length_814062_cov_49.488964-orf00057	-	-1.033318509	0.013429492

NODE_61_length_814062_cov_49.488964-orf00077	*gspL*	-2.082528478	0.011803031
NODE_61_length_814062_cov_49.488964-orf00094	-	-1.339415441	0.001851816
NODE_61_length_814062_cov_49.488964-orf00095	-	-1.105906382	0.016872465
NODE_61_length_814062_cov_49.488964-orf00162	-	-2.579678447	7.01E-07
NODE_61_length_814062_cov_49.488964-orf00163	-	-3.391219862	8.16E-11
NODE_61_length_814062_cov_49.488964-orf00164	-	-3.645979677	7.30E-07
NODE_61_length_814062_cov_49.488964-orf00165	*FlgN*	-1.817227215	4.35E-05
NODE_61_length_814062_cov_49.488964-orf00166	-	-1.490218837	0.000625681
NODE_61_length_814062_cov_49.488964-orf00168	*cheV*	-1.06445929	0.011904109
NODE_61_length_814062_cov_49.488964-orf00169	-	-1.009015432	0.018584845
NODE_61_length_814062_cov_49.488964-orf00171	*flgB*	-3.5699263	1.87E-09
NODE_61_length_814062_cov_49.488964-orf00172	*flgC*	-3.595894934	1.42E-09
NODE_61_length_814062_cov_49.488964-orf00173	*flgD*	-3.033169359	9.55E-10
NODE_61_length_814062_cov_49.488964-orf00174	*flgE*	-2.348390102	1.56E-07
NODE_61_length_814062_cov_49.488964-orf00176	*flgF*	-3.535860394	3.39E-11
NODE_61_length_814062_cov_49.488964-orf00177	*flgG*	-3.809972631	3.07E-12
NODE_61_length_814062_cov_49.488964-orf00178	*flgI*	-3.765657527	1.98E-10
NODE_61_length_814062_cov_49.488964-orf00179	*flgJ*	-3.373746092	1.87E-08
NODE_61_length_814062_cov_49.488964-orf00180	*flgH*	-3.836949629	2.89E-08
NODE_61_length_814062_cov_49.488964-orf00181	*flgK*	-2.273510146	3.21E-07
NODE_61_length_814062_cov_49.488964-orf00182	*flgL*	-1.887984107	1.86E-05
NODE_61_length_814062_cov_49.488964-orf00183	*ogt*	-1.073872858	0.012957894
NODE_61_length_814062_cov_49.488964-orf00185	-	-1.297348736	0.031103696
NODE_61_length_814062_cov_49.488964-orf00189	-	-1.196003017	0.006547409
NODE_61_length_814062_cov_49.488964-orf00190	-	-4.08349352	2.38E-18
NODE_61_length_814062_cov_49.488964-orf00191	-	-3.868553763	2.30E-10
NODE_61_length_814062_cov_49.488964-orf00199	*fliE*	-1.861781899	7.60E-05
NODE_61_length_814062_cov_49.488964-orf00200	*fliF*	-1.620280424	0.00027824
NODE_61_length_814062_cov_49.488964-orf00201	*fliG*	-1.37364275	0.004512479
NODE_61_length_814062_cov_49.488964-orf00203	*fliH*	-2.045866901	3.62E-05
NODE_61_length_814062_cov_49.488964-orf00204	*fliI*	-1.255474366	0.005640968
NODE_61_length_814062_cov_49.488964-orf00205	*fliJ*	-1.0153577	0.039567381
NODE_61_length_814062_cov_49.488964-orf00208	-	-1.834389839	0.000211002
NODE_61_length_814062_cov_49.488964-orf00209	*fliL*	-2.229756843	0.000115634
NODE_61_length_814062_cov_49.488964-orf00210	*fliM*	-1.185974565	0.009130414
NODE_61_length_814062_cov_49.488964-orf00211	*fliN*	-1.703551515	0.000560961
NODE_61_length_814062_cov_49.488964-orf00217	*flhA*	-4.324663749	3.36E-13
NODE_61_length_814062_cov_49.488964-orf00218	*flhF*	-3.630791347	6.09E-11
NODE_61_length_814062_cov_49.488964-orf00219	-	-2.279438951	4.04E-06
NODE_61_length_814062_cov_49.488964-orf00221	*fliA*	-1.392637227	0.005570857
NODE_61_length_814062_cov_49.488964-orf00222	*cheY*	-1.836814689	6.08E-05
NODE_61_length_814062_cov_49.488964-orf00223	*cheZ*	-1.887128506	2.42E-05
NODE_61_length_814062_cov_49.488964-orf00225	*cheA*	-1.797093856	4.49E-05
NODE_61_length_814062_cov_49.488964-orf00226	-	-1.75045364	0.000116681

NODE_61_length_814062_cov_49.488964-orf00227	-	-1.533735173	0.002916468
NODE_61_length_814062_cov_49.488964-orf00230	-	-1.974143246	4.13E-06
NODE_61_length_814062_cov_49.488964-orf00232	-	-1.584687286	0.000196419
NODE_61_length_814062_cov_49.488964-orf00233	*idhA*	-2.166156755	4.34E-07
NODE_61_length_814062_cov_49.488964-orf00234	*yidK*	-2.122265448	1.08E-06
NODE_61_length_814062_cov_49.488964-orf00236	-	-1.510833105	0.000657233
NODE_61_length_814062_cov_49.488964-orf00237	*dnaQ*	-1.075229099	0.019759328
NODE_61_length_814062_cov_49.488964-orf00240	-	-1.831545686	1.79E-05
NODE_61_length_814062_cov_49.488964-orf00241	*mocC*	-2.198260576	2.95E-07
NODE_61_length_814062_cov_49.488964-orf00242	-	-2.103915003	1.20E-06
NODE_61_length_814062_cov_49.488964-orf00251	-	-3.643489942	1.46E-11
NODE_61_length_814062_cov_49.488964-orf00268	-	-1.09108859	0.013613386
NODE_61_length_814062_cov_49.488964-orf00273	-	-1.17776742	0.028192521
NODE_61_length_814062_cov_49.488964-orf00354	*phlA*	-2.389513772	3.06E-06
NODE_61_length_814062_cov_49.488964-orf00355	*phlB*	-2.160054779	0.006072876
NODE_61_length_814062_cov_49.488964-orf00361	*mgtA*	-1.253876042	0.003196059
NODE_61_length_814062_cov_49.488964-orf00395	-	-5.120283849	2.21E-23
NODE_61_length_814062_cov_49.488964-orf00512	*napF*	-1.609955044	0.000302067
NODE_61_length_814062_cov_49.488964-orf00513	-	-1.461133436	0.026780029
NODE_61_length_814062_cov_49.488964-orf00514	*napA*	-2.338655063	0.000187233
NODE_61_length_814062_cov_49.488964-orf00515	*napA*	-3.097202962	1.33E-11
NODE_61_length_814062_cov_49.488964-orf00516	*napG*	-3.195627292	6.24E-12
NODE_61_length_814062_cov_49.488964-orf00518	*napH*	-2.910939576	2.68E-10
NODE_61_length_814062_cov_49.488964-orf00519	*napB*	-3.497847412	9.40E-14
NODE_61_length_814062_cov_49.488964-orf00520	*napC*	-3.567934229	1.04E-14
NODE_61_length_814062_cov_49.488964-orf00654	*garL*	-2.245008965	0.001111067
NODE_61_length_814062_cov_49.488964-orf00766	-	-1.664436485	0.020623756
NODE_61_length_814062_cov_49.488964-orf00822	*moaA*	-1.049106581	0.012641994
NODE_61_length_814062_cov_49.488964-orf00823	-	-1.097054009	0.040756224
NODE_61_length_814062_cov_49.488964-orf00824	*moaB*	-1.883465992	1.63E-05
NODE_61_length_814062_cov_49.488964-orf00826	*moaC*	-1.906290765	1.64E-05
NODE_61_length_814062_cov_49.488964-orf00827	*moaD*	-2.198109652	8.41E-05
NODE_61_length_814062_cov_49.488964-orf00828	*moaE*	-2.218141711	6.24E-06
NODE_61_length_814062_cov_49.488964-orf00830	*modA*	-1.796399444	9.00E-05
NODE_61_length_814062_cov_49.488964-orf00831	*modB*	-1.746268299	0.000443675
NODE_61_length_814062_cov_49.488964-orf00832	*modC*	-1.784316519	5.91E-05
NODE_61_length_814062_cov_49.488964-orf00865	*motA*	-3.035940485	1.14E-09
NODE_61_length_814062_cov_49.488964-orf00866	*motB*	-1.267653798	0.00382923
NODE_68_length_326006_cov_53.707497-orf00071	*srlR*	-1.334059165	0.001673408
NODE_68_length_326006_cov_53.707497-orf00072	-	-1.795880206	2.88E-05
NODE_68_length_326006_cov_53.707497-orf00080	*fliP*	-2.394909135	0.004849114
NODE_68_length_326006_cov_53.707497-orf00081	-	-2.945271473	0.001665133
NODE_68_length_326006_cov_53.707497-orf00082	*fliM*	-4.262303087	2.30E-08
NODE_68_length_326006_cov_53.707497-orf00084	-	-8.499732248	4.20E-09

NODE_68_length_326006_cov_53.707497-orf00085	*fliF*	-1.859439161	0.000293688
NODE_68_length_326006_cov_53.707497-orf00086	*fliG*	-1.796053164	0.00142216
NODE_68_length_326006_cov_53.707497-orf00088	*fliH*	-2.352885563	0.000266845
NODE_68_length_326006_cov_53.707497-orf00089	*fliI*	-2.767017101	2.89E-05
NODE_68_length_326006_cov_53.707497-orf00090	-	-2.434197458	0.001648699
NODE_68_length_326006_cov_53.707497-orf00095	*flgB*	-4.500591067	1.93E-09
NODE_68_length_326006_cov_53.707497-orf00096	-	-2.495302575	0.001197455
NODE_68_length_326006_cov_53.707497-orf00098	-	-4.176867767	0.000575412
NODE_68_length_326006_cov_53.707497-orf00099	*flgE*	-2.556005152	7.66E-06
NODE_68_length_326006_cov_53.707497-orf00100	*lfgF*	-2.447356259	7.11E-05
NODE_68_length_326006_cov_53.707497-orf00101	*lfgG*	-1.690474699	0.002821989
NODE_68_length_326006_cov_53.707497-orf00102	*lfgH*	-3.149516351	1.11E-06
NODE_68_length_326006_cov_53.707497-orf00104	*flgJ*	-1.51880873	0.012302827
NODE_68_length_326006_cov_53.707497-orf00105	*lfgK*	-2.039502626	3.83E-05
NODE_68_length_326006_cov_53.707497-orf00108	*fliC(lafA)*	-4.192453616	1.48E-17
NODE_68_length_326006_cov_53.707497-orf00110	*lafB*	-2.405982083	1.03E-06
NODE_68_length_326006_cov_53.707497-orf00111	*lafC*	-2.779792898	1.38E-06
NODE_68_length_326006_cov_53.707497-orf00112	*lafX*	-2.453772617	0.000315485
NODE_68_length_326006_cov_53.707497-orf00113	*lafE*	-4.481221027	3.93E-12
NODE_68_length_326006_cov_53.707497-orf00114	*lafF*	-2.499714783	5.51E-06
NODE_68_length_326006_cov_53.707497-orf00115	*fliA(lafS)*	-2.976959898	1.98E-07
NODE_68_length_326006_cov_53.707497-orf00116	*lafT*	-1.912930216	0.000105189
NODE_68_length_326006_cov_53.707497-orf00117	*lafU*	-1.20648241	0.008913313
NODE_68_length_326006_cov_53.707497-orf00130	-	-1.074924935	0.018566488
NODE_68_length_326006_cov_53.707497-orf00168	*ilvM*	-2.213726994	0.043991311
NODE_68_length_326006_cov_53.707497-orf00218	-	-1.502407839	0.002383913
NODE_68_length_326006_cov_53.707497-orf00219	-	-1.700975258	0.000886722
NODE_68_length_326006_cov_53.707497-orf00227	-	-1.722540804	0.045202178
NODE_68_length_326006_cov_53.707497-orf00240	-	-1.005983902	0.049644069
NODE_68_length_326006_cov_53.707497-orf00247	-	-1.418807303	0.001519132
NODE_68_length_326006_cov_53.707497-orf00317	*bioH*	-1.083538544	0.032467048
NODE_68_length_326006_cov_53.707497-orf00318	-	-1.789634138	0.000657824
NODE_69_length_373851_cov_50.515419-orf00092	-	-1.20376187	0.006418621
NODE_69_length_373851_cov_50.515419-orf00339	-	-1.007001786	0.02891159
NODE_69_length_373851_cov_50.515419-orf00341	*cysC*	-1.600273072	0.021443929
NODE_69_length_373851_cov_50.515419-orf00342	*cysN*	-1.333400163	0.008504992
NODE_69_length_373851_cov_50.515419-orf00343	*cysD*	-1.859754961	0.000296368
NODE_69_length_373851_cov_50.515419-orf00344	*cysG1*	-1.475959036	0.005227571
NODE_69_length_373851_cov_50.515419-orf00353	*cysI*	-1.491347666	0.001619109
NODE_69_length_373851_cov_50.515419-orf00356	-	-3.898810203	0.002133716
NODE_69_length_373851_cov_50.515419-orf00358	*cysJ*	-1.87739609	2.11E-05
NODE_69_length_373851_cov_50.515419-orf00381	-	-3.523848184	1.15E-10
NODE_69_length_373851_cov_50.515419-orf00443	-	-1.900932598	7.41E-05
NODE_7_length_42761_cov_75.093803-orf00003	-	-2.794173315	0.004849114

NODE_75_length_198257_cov_49.341919-orf00146	-	-2.075910839	0.000890608
NODE_75_length_198257_cov_49.341919-orf00158	*cueO*	-1.385798428	0.002573956
NODE_75_length_198257_cov_49.341919-orf00166	-	-1.504275879	0.012652254
NODE_75_length_198257_cov_49.341919-orf00190	-	-5.750207073	0.040964585
NODE_84_length_36912_cov_49.524055-orf00027	*rfe*	-1.192768237	0.004412395
NODE_10_length_17332_cov_72.932266-orf00009	-	1.013848866	0.041346057
NODE_10_length_17332_cov_72.932266-orf00010	-	1.108286955	0.016916751
NODE_125_length_1009_cov_73.777008-orf00001	-	1.316903737	0.015117999
NODE_146_length_604099_cov_50.948364-orf00201	*ompH_2*	1.444110545	0.000558019
NODE_146_length_604099_cov_50.948364-orf00249	-	1.46770137	0.007401946
NODE_146_length_604099_cov_50.948364-orf00318	*rumB*	1.03547707	0.048027933
NODE_146_length_604099_cov_50.948364-orf00338	*psrA*	1.839849929	5.01E-05
NODE_146_length_604099_cov_50.948364-orf00374	*sdhD*	1.288949942	0.007315276
NODE_146_length_604099_cov_50.948364-orf00377	*sdhC*	1.574996775	0.000232678
NODE_146_length_604099_cov_50.948364-orf00371	*sdhB*	1.818568757	0.012495401
NODE_146_length_604099_cov_50.948364-orf00372	*sdhA*	1.913468262	0.025475297
NODE_146_length_604099_cov_50.948364-orf00611	-	1.098173701	0.018855048
NODE_185_length_341402_cov_46.264301-orf00058	-	1.499335848	0.000590913
NODE_185_length_341402_cov_46.264301-orf00402	-	1.197709829	0.010928956
NODE_185_length_341402_cov_46.264301-orf00422	-	2.477363328	0.017862218
NODE_217_length_118346_cov_49.856361-orf00056	*suhB*	1.081953589	0.0106877
NODE_26_length_137541_cov_47.023979-orf00006	*nuoA*	1.107712448	0.009522545
NODE_26_length_137541_cov_47.023979-orf00007	*nuoB*	1.53017634	0.000280526
NODE_26_length_137541_cov_47.023979-orf00008	*nuoCD*	1.115203185	0.011328459
NODE_26_length_137541_cov_47.023979-orf00009	*nuoE*	1.593234269	0.003055706
NODE_26_length_137541_cov_47.023979-orf00010	*nuoF*	1.775398156	0.026105331
NODE_26_length_137541_cov_47.023979-orf00011	*nuoG*	1.477528337	0.033996117
NODE_26_length_137541_cov_47.023979-orf00012	*nuoH*	1.249882103	0.008590078
NODE_26_length_137541_cov_47.023979-orf00013	*nuoI*	2.477363328	0.017862218
NODE_26_length_137541_cov_47.023979-orf00014	*nuoJ*	1.135989205	0.012042712
NODE_26_length_137541_cov_47.023979-orf00016	*nuoK*	2.37151866	0.02704271
NODE_26_length_137541_cov_47.023979-orf00017	*nuoL*	1.370051128	0.001293767
NODE_26_length_137541_cov_47.023979-orf00018	*nuoM*	1.092581292	0.013782123
NODE_26_length_137541_cov_47.023979-orf00019	*nuoN*	1.024898826	0.014485426
NODE_61_length_814062_cov_49.488964-orf00067	*epsD*	1.477528337	0.042814164
NODE_61_length_814062_cov_49.488964-orf00116	-	1.859057739	0.002638872
NODE_61_length_814062_cov_49.488964-orf00337	-	2.143533041	0.008135331
NODE_61_length_814062_cov_49.488964-orf00441	-	1.017270454	0.023049899
NODE_61_length_814062_cov_49.488964-orf00521	*dppC*	1.056265721	0.024094788
NODE_61_length_814062_cov_49.488964-orf00586	-	1.444530771	0.022113468
NODE_61_length_814062_cov_49.488964-orf00696	*metR*	1.418079285	0.01408992
NODE_61_length_814062_cov_49.488964-orf00720	-	1.38151028	0.005944703
NODE_69_length_373851_cov_50.515419-orf00113	-	1.209415573	0.024345879
NODE_69_length_373851_cov_50.515419-orf00380	*hmpX*	1.157663182	0.001061935

NODE_69_length_373851_cov_50.515419-orf00403	-	2.109381983	0.004348298
NODE_7_length_42761_cov_75.093803-orf00041	-	1.044744418	0.00709646
NODE_75_length_198257_cov_49.341919-orf00023	-	5.49129475	0.020952111
NODE_75_length_198257_cov_49.341919-orf00139	*aceE*	1.093449086	0.049131109
NODE_75_length_198257_cov_49.341919-orf00143	*aceF*	1.031450675	0.040964585
NODE_75_length_198257_cov_49.341919-orf00144	*lpdA*	1.601512237	0.008490836
NODE_84_length_36912_cov_49.524055-orf00011	-	1.850265166	0.008204261

Gene id：Gene number

log2FoldChange: The ratio of the gene expression levels of the treatment to control groups then taken as the logarithm of base 2

Pvalue: The p-value of the significance test
